# ANP/GC-A signaling attenuates pulmonary metastasis of B16 melanoma enhanced by lipopolysaccharide or angiotensin-II

**DOI:** 10.1186/2050-6511-14-S1-P52

**Published:** 2013-08-29

**Authors:** Takashi Nojiri, Hiroshi Hosoda, Shin Ishikane, Toru Kimura, Kenji Kangawa

**Affiliations:** 1 Department of Biochemistry, National Cerebral and Cardiovascular Center Research Institute, Suita, Osaka, Japan; 2 Department of General Thoracic Surgery, Osaka University Graduate School of Medicine, Suita, Osaka, Japan

## Background

Systemic inflammation or activation of renin-angiotensin-aldosterone system (RAAS) play the essential role in the development of lifestyle-related disease. There is mounting evidence suggesting that systemic inflammation influences cancer growth, invasion, and metastasis. Furthermore, recent study has shown that activation of RAAS enhanced cancer metastasis.

Atrial natriuretic peptide (ANP) has been used clinically for the treatment with heart failure in Japan, and exhibits a wide range of cardioprotective effects, including antifibrosis, antihypertrophy, antiinflammation, and inhibition of RAAS through binding the guanylate cyclase-A (GC-A) receptor. This study was designed to examine whether ANP pretreatment attenuates pulmonary metastasis enhanced by lipopolysaccharide (LPS) or angiotensin-II.

## Materials and methods

We used B16 mice melanoma cell line, which don't have the GC-A receptor and angiotensin-II receptor, in the experimental hematogenous metastasis model in mice. We administered intravenously a dose of 1mg/kg LPS 4 hours before the injection of B16 cells, or angiotensin-II (subcutaneously via osmotic pump, 1mg/kg/min) 3 days before the injection of B16 cells.

ANP infusion (subcutaneously via osmotic-pump, 0.5μg/kg/min) was started one day before the injection of B16 cells. This dose does not change blood pressure and heart rate in mice.

## Results

Mice with LPS or angiotensin-II showed increasing of pulmonary metastasis compared to the control mice. The number of pulmonary metastasis of B16 melanoma (2.0 ×10^5^ cell/body) in 2 weeks was significantly lower ANP-pretreated mice compared to the control mice with LPS (62±12 vs. 248±36, P<0.001). Mice with angiotensin-II receptor blocker (Candesartan; oral, 8mg/kg/day) inhibited pulmonary metastasis of B16 melanoma enhanced by angiotensin-II. In addition, ANP-pretreated mice also showed the reduction of the number of pulmonary metastasis compared to the control mice with angiotensin-II.

## Conclusion

We found that ANP inhibited the induction of pulmonary metastasis by LPS or angiotensin-II. Our data provide novel insights into the prophylactic therapy in the cancer patients with lifestyle-related disease or during the perioperative period.

